# Impact of Booster Breaks and Computer Prompts on Physical Activity and Sedentary Behavior Among Desk-Based Workers: A Cluster-Randomized Controlled Trial

**DOI:** 10.5888/pcd13.160231

**Published:** 2016-11-17

**Authors:** Wendell C. Taylor, Raheem J. Paxton, Ross Shegog, Sharon P. Coan, Allison Dubin, Timothy F. Page, David M. Rempel

**Affiliations:** Author Affiliations: Raheem J. Paxton, School of Public Health and Institute of Healthy Aging, University of North Texas Health Science Center, Fort Worth, Texas; Ross Shegog, Sharon P. Coan, Allison Dubin, Department of Health Promotion and Behavioral Sciences, School of Public Health, The University of Texas Health Science Center at Houston, Houston, Texas; Timothy F. Page, Department of Health Policy and Management, Robert Stempel College of Public Health and Social Work, Florida International University, Miami, Florida; David M. Rempel, School of Medicine, University of California, San Francisco, California.

## Abstract

**Introduction:**

The 15-minute work break provides an opportunity to promote health, yet few studies have examined this part of the workday. We studied physical activity and sedentary behavior among office workers and compared the results of the Booster Break program with those of a second intervention and a control group to determine whether the Booster Break program improved physical and behavioral health outcomes.

**Methods:**

We conducted a 3-arm, cluster-randomized controlled trial at 4 worksites in Texas from 2010 through 2013 to compare a group-based, structured Booster Break program to an individual-based computer-prompt intervention and a usual-break control group; we analyzed physiologic, behavioral, and employee measures such as work social support, quality of life, and perceived stress. We also identified consistent and inconsistent attendees of the Booster Break sessions.

**Results:**

We obtained data from 175 participants (mean age, 43 y; 67% racial/ethnic minority). Compared with the other groups, the consistent Booster Break attendees had greater weekly pedometer counts (*P* < .001), significant decreases in sedentary behavior and self-reported leisure-time physical activity (*P* < .001), and a significant increase in triglyceride concentrations (*P* = .02) (levels remained within the normal range). Usual-break participants significantly increased their body mass index, whereas Booster Break participants maintained body mass index status during the 6 months. Overall, Booster Break participants were 6.8 and 4.3 times more likely to have decreases in BMI and weekend sedentary time, respectively, than usual-break participants.

**Conclusion:**

Findings varied among the 3 study groups; however, results indicate the potential for consistent attendees of the Booster Break intervention to achieve significant, positive changes related to physical activity, sedentary behavior, and body mass index.

## Introduction

The workplace presents opportunities for physical activity interventions, because a substantial percentage of US adults work in this setting, most of whom do not get the recommended levels of physical activity (1,2). Furthermore, sedentary workers have increased cardiovascular and metabolic risk and ultimately, premature mortality (3). Besides prolonged sitting during the workday, many employees eat unhealthy foods during the workday and have work-related stress (4). We theorized that promoting physical activity at work, particularly during work breaks, could be beneficial.

Despite their potential public health benefits to the workplace and despite workers’ desire for physical activity, only a few worksite physical activity interventions have been evaluated (2). A thorough review of the literature on short bouts of physical activity integrated into organizational routines yielded limited results; there is an absence of worksite studies related to physical activity that incorporate a comprehensive set of variables such as self-report and objective measures of physical activity, physiologic indices, sedentary behavior, and psychosocial and organizational factors (2). Because of these limitations, the Booster Break program was proposed, developed, and implemented to test the effects of a comprehensive worksite physical activity intervention (4–8). Booster Breaks are “organized, routine work breaks intended to improve physical and psychological health, enhance job satisfaction, and sustain or increase work productivity” (4). The Booster Break program uses discretionary paid time during the workday to make changes at the individual, environmental, and organizational levels (4–8).

We compared behavioral and health outcomes of 2 interventions — a group-based Booster Break intervention and an individual-based computer-prompt intervention — to those of a usual-break control group. We hypothesized that compared with participants in the other 2 study arms, Booster Break participants would have significant improvements in 1) physiologic measures (ie, blood pressure, fasting lipid and triglyceride concentrations, and anthropometrics); 2) physical activity (increase) and sedentary behavior (decrease); and 3) employee measures such as work social support, quality of life, and perceived stress.

## Methods

### Study design

We conducted a 3-arm, cluster-randomized controlled trial at 4 worksites in Texas from 2010 through 2013 to evaluate the Booster Break program. Worksite departments were assigned to 1 of 3 groups by using computerized random-number generation with an equal number of departments at each worksite represented in each group. We selected departments and jobs in which employees sat for at least 5 hours per day. Inclusion criteria were English proficiency, full-time employment (35–40 hrs/wk), being aged 18 years or older, and having no physician-specified limits on physical activity.

Interventions lasted 6 months. Data were collected from 2010 through 2013; data entry and analyses were conducted from 2014 through 2015. Participants were paid $25 for completing both baseline and follow-up assessments and received their results (weight, height, blood pressure, and cholesterol) from a free worksite health screening.

This study was approved by the Committee for the Protection of Human Subjects at The University of Texas Health Science Center and was registered in the ISRCTN Registry (no. ISRCTN2576399).

### Interventions

Both physical activity interventions were consistent with the World Health Organization’s Healthy Workplace Framework and Model, which strives to promote effective workplace interventions (9).

**Booster Break arm. **These structured, peer-led group sessions guided employees through a series of stretching, strengthening, and aerobic movements, followed by a 60-second meditation, described elsewhere (5). Daily worksite sessions lasted 13 to 15 minutes during one 15-minute break. Participants signed an attendance sheet.

**Computer-prompt arm.** This individualized intervention interrupted prolonged sedentary time by introducing 3-minute breaks at 5 hourly intervals daily, thus equaling the time used for the Booster Breaks (15 min). Each worksite installed computer software (Workrave version 1.10 [www.workrave.org], Eyes Relax version 0.87 [Centers for Disease Control and Prevention], and Compact Timer version 2.3.2896.29106 [S7, http://compact-timer-free-download.softwares7.com]) and provided training; prompts encouraged workers to get up and walk hallways, stairs, or outdoors. This intervention relies solely on each worker’s motivation, attention, and willingness to stop work when prompted. Participants completed a daily log in which they indicated whether they ignored, partially met, or fully met the computer-prompted physical activity breaks.

**Usual-break arm (control group).** This group practiced usual breaks without interventions at any level. Typical patterns were two 15-minute breaks (morning and afternoon) and 30 to 60 minutes for lunch. Previous studies found that usual-break practices rarely include health-promoting behaviors (eg, consuming nutritious foods, practicing meditation, performing any physical activity) (4,7,8).

### Measures

A team from the wellness services department of a local hospital traveled to each participating worksite at baseline and 6 months to complete physiologic assessments. Height, weight, waist circumference, and blood pressure measurements and blood sampling followed standard protocols to ensure validity and reliability (10–15). Fasting blood sampling was performed in the morning before employees started the workday. Lipid assessments (total cholesterol, high-density and low-density lipoprotein cholesterol, and triglycerides) were conducted at a certified laboratory compliant with Clinical Laboratory Improvement Amendments (http://wwwn.cdc.gov/clia/regulatory/default.aspx).

To assess objective levels of physical activity, we used step counts from New Lifestyles DigiWalker SW200 pedometers, which indicated movement, a measure of physical activity, following established protocols (16–18). The pedometer’s accuracy, reliability, and suitability for applied physical activity research are reported elsewhere (16–18). For one week at baseline and again at 6 months (program completion), each participant wore the pedometer each day — from rising in the morning until retiring at night — except when showering or bathing.

Physical activity was self-reported. We used the International Physical Activity Questionnaire long version (0.8 reported reliability and 0.3 criterion validity), which assesses moderate and vigorous physical activity in 5 domains, across which it has strong reliability and validity (19). It assesses time spent sitting — at work, at home, and during leisure time each day — as a measure of total sedentary time. We also used a scale that focuses exclusively on sedentary leisure time; it is a self-reported 7-day checklist, from the Neighborhood Quality of Life Study, that elicits data on the average daily number of minutes of leisure computer and Internet use, video games, telephone use, and television viewing. Its reliability and validity are reported elsewhere (20).

Employee and organizational constructs were work social support, quality of life, and perceived stress, which we assessed using the valid and reliable scales of Johnson et al (21), Ware et al (22), and Cohen and Williamson (23).

### Statistical analyses

Descriptive statistics were computed to characterize the study population. We used χ^2 ^tests of independence to identify any categorical differences in sociodemographic variables between study conditions. Subsequent nonparametric Kruskall–Wallis testing detected any mean or median differences in the continuous outcomes of the study at baseline. Generalized mixed-effects models (SAS Institute, Inc) were used to estimate within- and between-group changes. Because of normality concerns, we specified log normal or Poisson distributions in our mixed-effects models. Fixed effects in these analyses consisted of time, condition, a time-by-condition interaction (ie, between-group changes), and study covariates (age, race/ethnicity, and education). Participants nested within study conditions were treated as a random effect. If the time-by-condition interaction was significant, it was sliced to determine the relevance of within-group mean changes. For simplicity, we report adjusted least-square means and standard errors. All data were analyzed using SAS version 9.3 software (SAS Institute, Inc), and significance was set a *P* ≤ .05 with a 2-sided test.

**Posthoc analyses.** Our primary analysis led to additional questions that could help enhance the interpretation of our study. The questions related to 1) whether study outcomes differed by race/ethnicity, 2) whether intervention participants had better outcomes than usual-break participants, and 3) whether outcomes differed by program fidelity. We explored whether African American or Hispanic participants in the Booster Break and computer-prompt groups were more or less likely than non-Hispanic white participants to have improvements in quality of life, employee and lifestyle variables, sedentary behavior, or cardiometabolic markers. Adjusted logistic regression models were then used to determine whether the 2 intervention arms were more or less likely to make positive changes in study outcomes than the usual-break arm. Furthermore, changes in outcomes by consistency of participation in the Booster Break intervention were assessed to account for program fidelity, dose, and adherence. Departments considered to have consistent participation for the Booster Break sessions were defined as those with 80% or more of participants attending each session, an acceptable threshold to expect physiologic changes (5).

## Results

Participants (N = 185) were randomized by department (N = 35) to 1 of 3 treatment conditions: Booster Breaks (14 departments; 76 participants), computer prompts (9 departments, 61 participants), and usual breaks (12 departments, 48 participants) (Figure). Ten participants did not have any self-reported data at a given assessment and were eliminated from further analyses. Therefore, only 175 participants were used in the analysis. Participants were racially and ethnically diverse (35% African American, 33% non-Hispanic white, and 32% Hispanic) and had a mean age of 43 years; 82% were women, and 55% had a college degree or more (Table 1). The occupations were clerical (25%), nonclerical (55%), and managerial (ie, managers, supervisors, directors, and superintendents) (9%); information was missing for 11%. We found no significant differences in distribution by type of position (nonclerical vs clerical). We found differences in marital status, work social support, and body mass index (BMI) (all *P* = .02) by intervention condition (Table 2).

**Figure:**
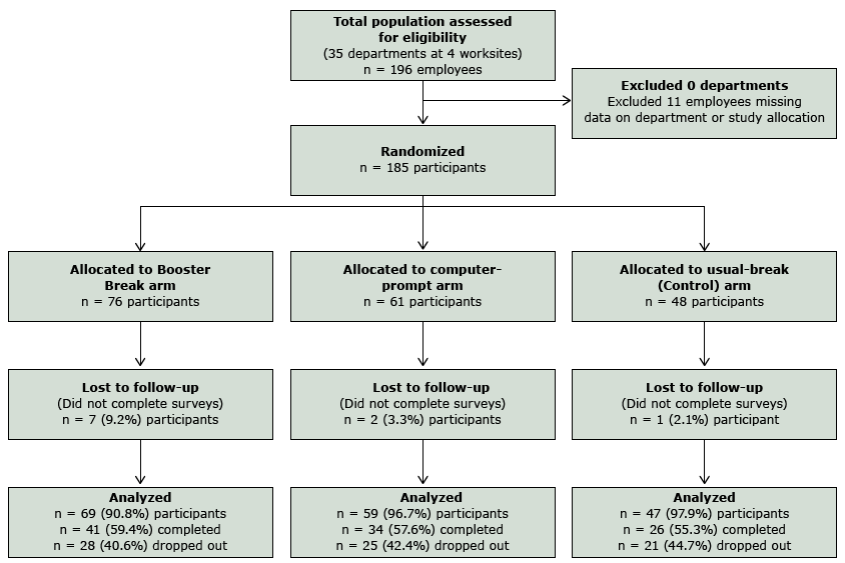
Stages of the Booster Break study for intent-to-treat analyses (all participants); 100% of participants were analyzed using intent-to-treat.

**Table 1:** Sociodemographic, Lifestyle, and Biological Characteristics of Study Participants (N = 175), by Study Completion Status, Texas, 2010–2013

Characteristic	Total (N = 175)	Completed (n = 101)	Not Completed (n =74)	*P* Value^a^
**Study arm, %**				
Usual breaks (control)	27	55	45	.91
Computer prompts	34	58	42	
Booster Breaks	39	59	41	
**Sociodemographics**				
**Age, mean (SD), y**	43.4 (11.9)	42.5 (12.0)	44.7 (12.1)	.21
**Married, %**	62	63	60	.72
**Race/ethnicity, %**				
Non-Hispanic white	33	58	42	.28
African American	35	49	51	
Hispanic	32	63	37	
**Education, %**				
High school diploma or less	9	73	27	.46
Some college	36	56	44	
College diploma or higher	55	57	43	
**Health-related quality of life scores, mean (SD)**				
Physical health^b^	56.8 (13.7)	57.3 (13.6)	56 (14.0)	.61
Mental health^c^	68.3 (16.9)	68.6 (18.1)	67.8 (15.1)	.15
Work social support^d^	3.9 (0.7)	4.0 (0.7)	3.9 (0.8)	.26
Perceived stress^e^	1.4 (0.6)	1.3 (0.6)	1.4 (0.7)	.55
**Lifestyle, mean (SD)**				
Body mass index, kg/m^2^	30.3 (7.4)	29.4 (8.0)	30.8 (6.7)	.17
Television viewing, min/wk	646 (543)	645 (514)	646 (586)	.59
LTPA MET, min/wk	612 (1,214)	709 (1,422)	480 (849)	.36
Total pedometer steps/wk	45,475 (21,144)	44,869 (23,280)	46,268 (18,122)	.17
**Selected cardiometabolic markers, mean (SD)**				
Total cholesterol, mg/dL	190 (35.1)	188 (35.4)	193 (34.8)	.32
Glucose, mg/dL	96 (27.7)	95.2 (32.0)	97 (20.3)	.39
Systolic blood pressure, mm Hg	116 (15.4)	114 (13.5)	120 (17.1)	.02

Abbreviations: LTPA, leisure-time physical activity; MET, metabolic equivalents; SD, standard deviation.

a *P *values for continuous variables were estimated with nonparametric testing; *P* values for categorical variables were estimated by χ^2^ testing for independence.

b Physical health was scored on a scale of 10 to 100, with higher scores indicating better health (22).

c Mental health was scored on a scale of 10 to 100, with higher scores indicating better health (22).

d Work social support was scored on a scale of 1 to 5, with higher scores indicating greater social support at work (21).

e Perceived stress was scored on a scale of 0 to 4, with higher scores indicating greater stress (23).

**Table 2:** Baseline Physiologic, Anthropometric, Lifestyle, and Psychosocial Characteristics of Study Participants (N = 175), by Study Arm, Texas, 2010–2013

Characteristic	Usual Breaks (n = 47)	Computer Prompts (n = 59)	Booster Breaks (n = 69)	*P* Value^a^
**Sociodemographics**				
**Married, %**	63	48	72	.02
**Race/ethnicity, %**				
Non-Hispanic white	27	42	31	.30
African American	28	24	48	
Hispanic	26	33	41	
**Education, %**				
High school diploma or less	15	3	9	.07
Some college	25	34	45	
College diploma or higher	60	63	46	
**Sociodemographics, median (LCL^b^, UCL^c^)**				
Age, y	42 (32, 49)	44 (35, 56)	43 (36, 52)	.26
Weight, lb	186 (162, 211)	163 (152, 173)	197 (176, 218)	.08
Body mass index, kg/m^2^	29.8 (27.7, 31.8)	28.3 (26.8, 29.9)	32.2 (30.2, 34.3)	.02
Waist circumference, in	39. 4 (37.2, 41.6)	36.9 (35.5, 38.3)	39.9 (37.7, 42.1)	.11
LTPA MET, min/wk	99 (0, 396)	421 (0, 975)	99 (0, 767)	.11
Total pedometer steps/wk	43,558 (37,693, 49,424)	46,860 (41,311, 52,408)	45,633 (39,335, 51,932)	.60
**Sedentary time**				
Computer use, min/wk	791 (316, 1,267)	293 (138, 448)	1,075 (453, 1,697)	.11
Television viewing, min/wk	736 (558, 915)	670 (475, 864)	813 (436, 1,189)	.48
Sedentary min/weekday	3,069 (1,801, 4,338)	2,917 (2,193, 3,641)	3,226 (2,336, 4,117)	.74
Sedentary min/weekend	950 (426, 1,473)	602 (464, 740)	794 (620, 969)	.14
**Health-related quality-of-life scores, median (LCL^b^, UCL^c^)**				
Physical health^d^	82.3 (76.6, 87.9)	83.4 (78.9, 87.7)	81.2 (76.7, 85.6)	.72
Mental health^e^	77.2 (71.4, 82.9)	76.7 (71.8, 81.6)	75.3 (70.9, 79.7)	.75
Work social support^f^	3.9 (3.7, 4.1)	4.1 (4.0, 4.3)	3.8 (3.6, 3.9)	.02
Perceived stress^g^	1.2 (1.0, 1.4)	1.3 (1.1, 1.5)	1.5 (1.3, 1.6)	.10
**Cardiometabolic markers, median (LCL^b^, UCL^c^)**				
Total cholesterol, mg/dL	191 (184, 199)	182 (175, 190)	196 (185, 207)	.15
High-density lipoprotein cholesterol, mg/dL	52 (47, 57)	48 (45, 51)	53 (50, 56)	.12
Low-density lipoprotein cholesterol, mg/dL	110 (102, 119)	108 (99, 116)	114 (105, 124)	.58
Triglycerides, mg/dL	121 (97, 144)	106 (89, 123)	121 (92, 150)	.82
Blood glucose, mg/dL	94 (89, 99)	94 (90, 98)	99 (89, 108)	.84
Systolic blood pressure, mm Hg	113 (109, 118)	116 (112, 119)	119 (115, 123)	.41
Diastolic blood pressure, mm Hg	70 (67, 73)	70.0 (67, 73)	71 (69, 73)	.83

Abbreviations: LCL, lower confidence limit; LTPA leisure-time physical activity; MET, metabolic equivalent; UCL, upper confidence limit.

a *P *values for continuous variables were estimated with nonparametric testing; *P* values for categorical variables were estimated by χ^2^ testing for independence.

b Lower confidence limit = 25%.

c Upper confidence limit = 75%.

d Physical health was scored on a scale of 10 to 100, with higher scores indicating better health (22).

e Mental health was scored on a scale of 10 to 100, with higher scores indicating better health (22).

f Work social support was scored on a scale of 1 to 5, with higher scores indicating greater social support at work (21).

g Perceived stress was scored on a scale of 0 to 4, with higher scores indicating greater stress (23).

Attrition (or drop out) was estimated at 42% overall and was not significantly different among the 3 arms. Attrition did not differ by sociodemographic, quality-of-life, behavioral, or cardiometabolic variables, with one exception: baseline mean systolic blood pressure was greater among those lost to follow-up (*P* = .02) (Table 1).

On average, African Americans had significantly higher BMIs (*P* = .003), higher systolic blood pressure (*P* <.001), higher diastolic blood pressure (*P* < .001), and larger waist circumference (*P* = .04) than other participants. Also, African Americans reported significantly more sedentary time on weekends (*P* = .045) and while using the computer (*P* = .03) and watching television (*P* = .004). Hispanic participants had higher serum triglyceride concentrations than other participants (*P* = .007); non-Hispanic whites reported significantly more leisure-time physical activity (*P* = .02) and work social support (*P* = .002) than other participants.

### Changes over time by study condition: a priori hypotheses — intent-to-treat analysis

No significant time-by-condition (baseline to change) interactions or main effects were observed among quality-of-life variables in any of the 3 study arms (Table 3). The time-by-condition interaction for waist circumference was significant (*P* = .05). We found a small increase in waist circumference among computer-prompt participants (*P* = .047) but no significant change among Booster Break (*P* = .29) or usual-break (*P* = .33) participants. Other anthropometric variables did not differ significantly. Significant time-by-condition interactions were observed for pedometer counts (*P* = .001) and metabolic-equivalent (MET) minutes of physical activity (*P* = .001). Weekly pedometer counts increased among usual-break participants (*P* < .001) but decreased among computer-prompt participants (*P* < .001) and Booster Break participants (*P* < .001). Similar results were observed for average daily pedometer counts. Leisure-time physical activity per week increased for usual-break (*P* < .001) and computer-prompt (*P* < .001) participants but decreased for Booster Break participants (*P* < .001). Significant time-by-condition interactions were observed for computer use (*P* = .001), television viewing (*P* = .001), and both weekday (Monday through Friday, *P* < .001) and weekend (Saturday through Sunday, *P* < .001) sedentary time. Computer use decreased for Booster Break participants (*P* < .001), decreased for usual-break participants (*P* = .04), and increased for computer-prompt participants (*P* < .001). Television viewing time decreased among all study participants, with the greatest reductions in usual-break (*P* < .001), compared with computer-prompt (*P* < .001) and Booster Break (*P* < .001) participants. Weekday sedentary behavior increased significantly for usual-break participants (*P* < .001) and Booster Break participants (*P* = .04) but not for computer-prompt participants (*P* = .20). Weekend sedentary behavior decreased for computer-prompt and Booster Break participants (both *P* < .001) but did not change significantly for usual-break participants (*P* = .61). A significant time-by-condition interaction was observed for serum triglyceride concentrations, which increased among computer-prompt (*P* < .001) and Booster Break (*P* = .001) but not among usual-break (*P* = .61) participants.

**Table 3:** Comparison of Baseline and Follow-up Characteristics of Study Participants (N = 175), by Study Arm, Texas, 2010–2013^a^

Variable	Usual Breaks (n = 47)		Computer Prompts (n = 59)		Booster Breaks (n = 69)		Time by Group *P* Value^b^
	Baseline	Follow-up	Baseline	Follow-up	Baseline	Follow-up	
**Health-related quality-of-life scores**							
Physical health^c^	58.1 (2.2)	61.3 (2.2)	56.7 (2.5)	60.0 (2.5)	59.1 (2.0)	59.7 (2.0)	.33
Mental health^d^	72.4 (3.0)	68.2 (3.0)	70.2 (2.8)	71.0 (2.8)	70.7 (2.6)	70.5 (2.6)	.11
Work social support e	3.8 (0.1)	3.9 (0.1)	4.0 (0.1)	3.9 (0.1)	3.7 (0.1)	3.6 (0.1)	.38
Perceived stress^f^	1.1 (0.1)	1.2 (0.1)	1.3 (0.1)	1.2 (0.1)	1.4 (0.1)	1.4 (0.1)	.59
**Anthropometrics**							
Body mass index, kg/m^2^	29.1 (1.1)	29.3 (1.1)	27.9 (1.1)	28.1 (1.1)	31.1 (1.3)	31.0 (1.3)	.97
Waist circumference, in	38.6 (1.2)	38.4 (1.2)	36.2 (1.0)	36.5 (1.0)^g^	38.6 (1.3)	38.4 (1.3)	.05
Weight, lb	182.7 (8.5)	183.3 (8.5)	167.0 (8.4)	169.1 (8.4)^h^	183.2 (7.9)	183.3 (7.9)	.22
**Physical activity**							
Total pedometer steps/wk	47,591 (4,354)	47,856 (4,345)^h^	53,444 (4,346)	53,113 (4,229)^h^	49,514 (4,058)	47,341 (4,040)^h^	.001
Average pedometer steps/d	6,822 (617)	6,904 (615)^h^	7,836 (603)	7,625 (599)^h^	7,176 (575)	6,834 (572)^h^	.001
LTPA METs	326 (134)	362 (134)^h^	647 (156)	683 (156)^h^	726 (218)	666 (218)^h^	.001
**Sedentary behavior**							
Computer usage, min/wk	570 (111)	556 (111)	398 (95)	555 (94)^g^	644 (99)	406 (100)^h^	.001
Television viewing, min/wk	719 (92)	648 (92)^h^	674 (95)	632 (94)^h^	652 (85)	625 (85)^h^	.001
Sedentary min/weekday	3,416 (777)	3,978 (777)^h^	3,392 (521)	3,405 (521)	3,497 (571)	3,516 (570)^g^	<.001
Sedentary min/weekend	984 (267)	987 (267)	676 (90)	614 (90)^h^	809 (97)	755 (97)^h^	<.001
**Cardiometabolic markers**							
Total cholesterol, mg/dL	197 (4.8)	197 (4.8)	185 (5.3)	191 (5.3)^g^	199 (6.1)	200 (6.1)^h^	.21
High-density lipoprotein cholesterol, mg/dL	52 (2.6)	53 (2.6)	48 (2.2)	40 (2.2)	53 (2.2)	55 (2.2)^h^	.80
Low-density lipoprotein cholesterol, mg/dL	120 (5.2)	122 (5.2)	114 (5.7)	123 (5.7)^h^	121 (5.8)	123 (5.8)	.09
Triglycerides, mg/dL	126 (12.8)	126 (12.8)	104 (12.2)	112 (12.2)^h^	121 (16.5)	128 (16.5)^h^	.02
Glucose, mg/dL	96 (3.3)	95 (3.3)	95 (3.0)	94 (3.0)	98 (5.5)	99 (5.5)	.60
Diastolic blood pressure, mm Hg	69 (1.7)	70 (1.7)	70 (1.5)	72 (1.5)	70 (1.3)	72 (1.3)	.84
Systolic blood pressure, mm Hg	115 (2.5)	118 (2.5)	118 (2.2)	123 (2.2)^g^	118 (2.4)	120 (2.4)	.59

Abbreviations: LTPA, leisure-time physical activity; MET, metabolic equivalent.

a Values are mean (standard error) unless otherwise indicated. An intent-to-treat analysis was applied when participants were followed, regardless of adherence to program.

b * P* values calculated by mixed effects models testing the effects of time, condition, time by condition and adjusted for age, education, and race/ethnicity.

c Physical health was scored on a scale of 10 to 100, with higher scores indicating better health (22).

d Mental health was scored on a scale of 10 to 100, with higher scores indicating better health (22).

e Work social support was scored on a scale of 1 to 5, with higher scores indicating greater social support at work (21).

f Perceived stress was scored on a scale of 0 to 4, with higher scores indicating greater stress (23).

g *P* < .05.

h *P* < .01.

### Posthoc analyses

Logistic regression models adjusted for age and education indicated that African American and Hispanic participants were no more or less likely than non-Hispanic white participants to make improvements in study outcomes. Adjusted logistic regression models indicated that Booster Break participants were 6.8 and 4.3 times more likely to have decreases in BMI and weekend sedentary time, respectively, than usual-break participants.

Differences in outcomes by consistency of participation in the Booster Break intervention showed that overall, inconsistent attendees were less likely to be married (*P* = .006) and more likely to be African American (*P* < .001) or to report lower mean levels of work social support (*P* = .003) than consistent attendees. Additionally, inconsistent attendees had significantly greater serum concentrations of glucose (*P* < .001) and triglycerides (*P* = .009) and higher systolic and diastolic blood pressure (*P* < .001) than consistent attendees (Table 4).

**Table 4:** Sociodemographic, Lifestyle, and Biological Characteristics of Study Participants (N = 175), by Intervention Attendance Status, Texas, 2010–2013^a^

Variable	Inconsistent Attendance (n = 69)	Consistent Attendance (n = 106)	*P* Value^b^
**Sociodemographics**			
**Married, %**	53	67	.006
**Race/ethnicity, %**			
Non-Hispanic white	24	39	<.001
African American	52	24	
Hispanic	24	38	
**Education, %**			
High school diploma or less	7	9	.48
Some college	33	38	
College diploma or higher	60	53	
**Age, median (LCL^c^, UCL^d^), y**	43 (35, 52)	44 (34, 52)	.80
**Health-related quality of life scores, median (LCL^c^, UCL^d^)**			
Physical health^e^	58 (54, 63)	60 (54, 63)	.51
Mental health^f^	75 (64, 80)	72 (60, 80)	.55
Work social support^g^	3.8 (3.5, 4.0)	4.0 (3.8, 4.5)	.003
Perceived stress^h^	1.2 (0.8, 1.9)	1.3 (1.0, 1.8)	.48
**Lifestyle characteristics,** **median (LCL^c^, UCL^d^) **			
Body mass index, kg/m^2^	29.1 (25.1, 32.9)	28.7 (25.1, 35.2)	.95
Waist circumference, in	37.5 (35.4, 40.7)	37.0 (33.3, 43.3)	.65
Weight, lb	178.9 (156.0, 213.3)	168.7 (146.4, 214.3)	.33
Total pedometer steps/wk	43,325 (32,498, 58,216)	40,709 (30,708, 52,739)	.24
Average pedometer steps/d	6,302 (4,991, 8,389)	5,816 (4,387, 7,575)	.12
LTPA METs	198 (0, 657)	198 (0, 918)	.89
Computer use, min/wk	150 (60, 450)	120 (40, 420)	.64
Television viewing, min/wk	630 (270, 840)	420 (240, 840)	.40
Sedentary min/weekday	2,400 (1,800, 3,000)	2,250 (1,800, 2,925)	.57
Sedentary min/weekend	480 (360, 960)	600 (360, 960)	.94
**Cardiometabolic markers, median (LCL^c^, UCL^d^)**			
Total cholesterol, mg/dL	186 (168, 213)	185 (163, 211)	.54
High-density lipoprotein cholesterol, mg/dL	50 (42, 60)	50 (41, 57)	.66
Low-density lipoprotein cholesterol, mg/dL	109 (88, 137)	109 (83, 132)	.66
Glucose, mg/dL	98 (89, 109)	97 (82, 94)	<.001
Triglycerides, mg/dL	102 (78, 140)	85 (65, 124)	.009
Systolic blood pressure, mm Hg	119 (108, 127)	112 (104, 121)	<.001
Diastolic blood pressure, mm Hg	73 (68, 127)	68 (62, 75)	<.001

Abbreviations: LCL, lower confidence limit; LTPA leisure-time physical activity; MET, metabolic equivalent; UCL, upper confidence limit.

a Departments considered to have consistent participation for the Booster Break sessions were those with 80% or more of participants attending each session.

b *P* values for continuous variables were estimated with nonparametric testing; *P* values for categorical variables were estimated by χ^2^ testing for independence.

c Lower confidence limit = 25%.

d Upper confidence limit = 75%.

e Physical health was scored on a scale of 10 to 100, with higher scores indicating better health (22).

f Mental health was scored on a scale of 10 to 100, with higher scores indicating better health (22).

g Work social support was scored on a scale of 1 to 5, with higher scores indicating greater social support at work (21).

h Perceived stress was scored on a scale of 0 to 4, with higher scores indicating greater stress (23).

### Changes over time by attendance status: intent-to-treat analysis

Consistent Booster Break participants had significant time-by-condition interactions for BMI (*P* = .049), weekly pedometer counts (*P* < .001), MET-minutes of physical activity (*P* < .001), computer usage (*P* < .001), television viewing (*P* < .001), serum triglycerides (*P* = .02), and both weekday (*P* < .001) and weekend sedentary (*P* < .001) behavior (Table 5). BMI increased significantly among the usual-break participants (*P* = .02) but did not significantly change among the remaining participants (computer prompts, *P* = .58; Booster Breaks, *P* = .49). Weekly pedometer counts increased among Booster Break (*P* < .001) and decreased among computer-prompt (*P* < .001) and usual-break (*P* < .001) participants. Weekly MET-minutes of physical activity increased among usual-break (*P* < .001) and computer-prompt (*P* < .001) but decreased among Booster Break (*P* < .001) participants. Computer use decreased among Booster Break (*P* < .001) participants, but increased among usual-break (*P* < .001) and computer-prompt (*P* < .001) participants. Television viewing time decreased significantly among all groups, with the greatest decrease among Booster Break (*P* < .001) participants. Weekday sedentary time increased among all participants (all *P* < .001), with the greatest increase observed among usual-break (*P* < .001) participants. Significant reductions in weekend sedentary time were observed among Booster Break (*P* < .001) and computer-prompt (*P* = .003) participants, whereas no changes were observed among usual-break (*P* = .07) participants. Lastly, serum triglyceride concentrations increased significantly among Booster Break (*P* < .001) and computer-prompt (*P* = .005) participants but did not change significantly for usual-break (*P* = .06) participants (Table 5).

**Table 5:** Physiologic, Anthropometric, Lifestyle, and Psychosocial Characteristics of Consistent Study Attendees^a^ (N = 106), Texas, 2010–2013^b^

Variable	Usual Breaks (n = 30)		Computer Prompts (n = 39)		Booster Breaks (n = 37)		Time by Group *P* Value^c^
	Baseline	Follow-up	Baseline	Follow-up	Baseline	Follow-up	
**Health-related quality-of-life scores**							
Physical health^d^	59.0 (3.2)	60.0 (3.2)	57.4 (3.5)	61.7 (3.5)	58.9 (3.0)	60.2 (3.0)	.66
Mental health^e^	71.1 (4.6)	67.8 (4.6)	72.5 (4.7)	72.0 (4.6)	70.1 (4.3)	69.5 (4.3)	.56
Work social support^f^	3.8 (0.1)	3.9 (0.1)	4.1 (0.2)	4.0 (0.2)	3.8 (0.2)	3.6 (0.2)	.14
Perceived stress^g^	1.2 (0.2)	1.3 (0.2)	1.2 (0.1)	1.1 (0.1)	1.3 (0.1)	1.3 (0.1)	.77
**Lifestyle characteristics**							
Body mass index, kg/m^2^	28.2 (1.9)	29.2 (1.9)^h^	27.7 (1.9)	27.9 (1.9)	31.4 (1.8)	31.0 (1.8)	.049
Waist circumference, in	38.6 (1.9)	38.5 (1.9)	36.4 (1.9)	36.6 (1.9)	39.4 (1.8)	38.7 (1.8)^h^	.08
Weight, lb	187.5 (13.0)	189.3 (13.0)	169.8 (13.7)	171.6 (13.7)	193.6 (12.4)	192.4 (12.4)	.16
Total pedometer steps/wk	50,713 (5,461)	48,780 (5,462)^i^	59,378 (7,563)	57,811 (7,542)^i^	45,023 (5,139)	46,689 (5,113)^i^	<.001
Average pedometer steps/d	7,250 (781)	6,973 (781)	8,577 (1,081)	8,299 (1,079)	6,465 (731)	6,754 (727)	.45
LTPA METs	252 (114)	284 (114)^i^	891 (198)	934 (198)^i^	658 (188)	601 (188)^i^	<.001
**Sedentary behavior**							
Computer use, min/wk	349 (132)	372 (131)^i^	258 (132)	437 (132)^i^	737 (147)	383 (147)^i^	<.001
Television viewing, min/wk	707 (122)	683 (122)^i^	767 (127)	725 (126)^i^	804 (114)	743 (114)^i^	<.001
Sedentary min/weekday	2,285 (559)	2,960 (559)^i^	3,208 (638)	3,307 (638)^i^	3,415 (621)	3,598 (618)^i^	<.001
Sedentary min/weekend	629 (165)	617 (165)	535 (138)	519 (138)^h^	819 (141)	681 (141)^i^	<.001
**Cardiometabolic markers**							
Total cholesterol, mg/dL	209 (7.1)	211 (7.1)	196 (8.1)	204 (8.1)^h^	211 (8.8)	214 (8.8)	.33
High-density lipoprotein cholesterol, mg/dL	48 (3.8)	50 (3.8)	48 (3.6)	51 (3.6)	50 (3.6)	52 (3.6)	.88
Low-density lipoprotein cholesterol, mg/dL	135 (7.7)	139 (7.7)	126 (8.9)	137 (8.9)^i^	135 (9.2)	140 (9.2)^h^	.12
Triglycerides, mg/dL	131 (19.3)	137 (19.3)	131 (17.0)	137 (19.2)^i^	119 (18.7)	135 (18.7)^i^	.02
Blood glucose, mg/dL	94 (5.0)	96 (5.0)	97 (5.3)	97 (5.3)	98 (9.1)	100 (9.1)	.78
Systolic blood pressure, mm Hg	117 (4.0)	121 (4.0)	117 (3.7)	124 (3.7)^h^	118 (3.7)	124 (3.7)^h^	.67
Diastolic blood pressure, mm Hg	70 (2.3)	73 (2.3)	70 (2.4)	74 (2.4)^i^	69 (2.1)	74 (2.1)^i^	.38

Abbreviations: LTPA, leisure time physical activity; MET, metabolic equivalents.

a Departments considered to have consistent participation for the Booster Break sessions were those with 80% or more of participants attending each session.

b Values are mean (standard error) unless otherwise indicated.

c *P* values calculated by mixed-effects models testing the effects of time, condition, time by condition and adjusted for age, education, and race/ethnicity.

d Physical health was scored on a scale of 10 to 100, with higher scores indicating better health (22).

e Mental health was scored on a scale of 10 to 100, with higher scores indicating better health (22).

f Work social support was scored on a scale of 1 to 5, with higher scores indicating greater social support at work (21).

g Perceived stress was scored on a scale of 0 to 4, with higher scores indicating greater stress (23).

h *P* < .05

i *P* < .01.

Inconsistent attendees had significant time-by-group interactions for physical health (*P* = .02); weekly pedometer counts (*P* < .001); MET-minutes of physical activity (*P* < .001), computer usage (*P* < .001), television viewing (*P* < .001), and weekday and weekend sedentary behavior (*P* < .001); and serum triglyceride concentrations (*P* = .001) (Table 6). Physical health increased among usual-break (*P* = .001) but not among computer-prompt (*P* = .71) or Booster Break (*P* = .93) participants. Weekly pedometer counts increased among usual-break (*P* < .001) but decreased among computer-prompt (*P* < .001) and Booster Break (*P* < .001) participants. Similarly, weekly MET-minutes of physical activity increased among usual-break (*P* < .001) and computer-prompt (*P* = .001) but decreased among Booster Break (*P* < .001) participants. Computer usage decreased among usual-break (*P* < .001) and Booster Break (*P* < .001) but increased among computer-prompt (*P* < .001) participants. Television viewing decreased among usual-break (*P* < .001) and computer-prompt (*P* < .001) participants, but no change was observed among Booster Break (*P* = .08) participants. Weekday sedentary behavior significantly decreased among Booster Break (*P* < .001) and computer-prompt (*P* < .001) but increased significantly among usual-break (*P* < .001) participants. Weekend sedentary behavior increased significantly among usual-break (*P* = .02) and Booster Break (*P* < .001) but decreased among computer-prompt (*P* < .001) participants. Lastly, serum triglyceride concentrations decreased among usual-break (*P* = .008) participants, increased significantly among computer-prompt (*P* = .01) participants, and did not change significantly among Booster Break (*P* = .16) participants (Table 6).

**Table 6:** Physiologic, Anthropometric, Lifestyle, and Psychosocial Characteristics of Inconsistent Study Attendees^a^ (N = 69), Texas, 2010–2013^b^

Characteristic	Usual Breaks (n = 17)		Computer Prompts (n = 20)		Booster Breaks (n = 32)		Time-by-Group P Value^c^
	Baseline	Follow-up	Baseline	Follow-up	Baseline	Follow-up	
**Health-related quality-of-life scores**							
Physical health^d^	56.5 (4.5)	63.7 (4.5)^e^	55.2 (3.9)	56.1 (3.9)	60.6 (3.1)	60.5 (3.1)	.02
Mental health^f^	77.0 (4.7)	71.1 (4.7)^g^	66.7 (4.1)	70.2 (4.1)	73.7 (3.6)	74.2 (3.6)	.06
Work social support^h^	4.0 (0.1)	3.9 (0.1)	3.7 (0.2)	3.8 (0.2)	3.6 (0.2)	3.5 (0.2)	.29
Perceived stress^i^	1.0 (0.2)	1.0 (0.2)	1.4 (0.2)	1.3 (0.2)	1.4 (0.2)	1.4 (0.2)	.60
**Lifestyle characteristics**							
Body mass index, kg/m^2^	30.2 (1.8)	29.0 (1.8)^g^	27.2 (1.5)	27.4 (1.5)	30.9 (2.1)	31.0 (2.1)	.07
Waist circumference, in	39.0 (2.1)	38.6 (2.1)	35.8 (1.5)	36.5 (1.5)^g^	38.5 (2.2)	38.8 (2.2)	.06
Weight, lb	184.0 (13.9)	182.3 (13.8)	170.3 (13.1)	172.9 (13.1)^g^	178.6 (11.6)	180.2 (11.6)	.16
Total pedometer steps/wk	48,848 (6,202)	52,586 (6,202)^e^	52,207 (6,135)	51,669 (6,135)^e^	55,353 (5,038)	50,055 (5,038)^e^	<.001
Average pedometer steps/d	7,037 (878)	7,687 (878)	7,754 (810)	7,344 (810)	8,088 (702)	7,229 (702)	.48
LTPA METs	664 (282)	706 (281)^e^	287 (228)	309 (228)^e^	808 (408)	744 (408)^e^	<.001
**Sedentary behavior**							
Computer use, min/week	803 (235)	721 (235)^e^	380 (163)	486 (163)^e^	443 (129)	354 (129)^e^	<.001
Television viewing, min/wk	766 (187)	610 (187)^e^	570 (189)	539 (189)^e^	510 (154)	526 (154)	<.001
Sedentary min/weekday	4,563 (2,013)	4,913 (2,013)^e^	3,102 (1,026)	2,952 (1,026)^e^	3,156 (1,083)	2,993 (1,083)^e^	<.001
Sedentary min/weekend	1,483 (721)	1,515 (721)^g^	895 (167)	731 (167)^e^	723 (164)	772 (164)^e^	<.001
**Cardiometabolic markers**							
Total cholesterol, mg/dL	187 (7.6)	185 (7.6)	187 (8.6)	189 (8.6)	190 (9.1)	188 (9.1)	.71
High-density lipoprotein cholesterol, mg/dL	59 (4.5)	58 (4.5)	45 (3.6)	45 (3.6)	56 (3.3)	58 (3.3)	.73
Low-density lipoprotein cholesterol, mg/dL	110 (8.0)	109 (8.0)	115 (8.9)	118 (9.0)	113 (7.6)	112 (7.6)	.46
Triglycerides, mg/dL	108 (20.7)	98 (20.7)^e^	131 (22.1)	140 (22.1)^g^	105 (31.0)	100 (31.0)	.001
Blood glucose, mg/dL	97 (8.9)	89 (6.9)^g^	93 (5.3)	90 (5.3)	97 (6.5)	97 (6.5)	.25
Systolic blood pressure, mm Hg	112 (3.9)	115 (3.9)	121 (3.8)	122 (3.8)^e^	117 (3.6)	115 (3.6)^g^	.60
Diastolic blood pressure, mm Hg	70 (2.9)	69 (2.9)	72 (2.4)	71 (2.4)	71 (1.9)	70 (1.9)	>.99

Abbreviations: LTPA, leisure time physical activity; MET, metabolic equivalent.

a Departments considered to have inconsistent participation for the Booster Breaks sessions were those with fewer than 80% or more of participants attending each session.

b Values are mean (standard error) unless otherwise indicated.

c *P* Values calculated by mixed-effects models testing the effects of time, condition, time by condition and adjusted for age, education, and race/ethnicity.

d Physical health was scored on a scale of 10 to 100, with higher scores indicating better health (22).

e *P* < .01.

f Mental health was scored on a scale of 10 to 100, with higher scores indicating better health (22).

g *P* < .05.

h Work social support was scored on a scale of 1 to 5, with higher scores indicating greater social support at work (21).

i Perceived stress was scored on a scale of 0 to 4, with higher scores indicating greater stress (23).

## Discussion

This trial evaluated the effects of the Booster Break and computer-prompt interventions among a racially and ethnically diverse population by using a complex and novel research design. The results varied and yielded no clear patterns for the a priori hypotheses analyses or for the posthoc analyses of inconsistent attendees of the Booster Break program. As a result of meeting the performance criteria of 80% attendance, the consistent attendees of the Booster Break program received a sufficient dose of the intervention to assess changes. Consistent attendees in the Booster Break study arm increased their weekly pedometer counts and decreased their sedentary behavior as well as maintained BMI status whereas the usual-break group significantly increased their BMI. In both intervention groups, triglyceride concentrations increased although remained within the normal range.

The Booster Break intervention is a simple, peer led, 15-minute group-based physical activity performed at work, in work attire, without equipment, and during standard breaks. Despite this convenience and simplicity, having all participants attend the sessions at a high, sustained level was challenging. It is noteworthy that consistent attendees reported having significantly greater work social support than inconsistent attendees reported. This finding may indicate unique and fundamental differences among the worksites related to readiness and receptivity for health promotion initiatives.

Because participants were predominantly female, selection bias and the generalizability of our findings to populations of men, other professions, and nonvolunteers are unknown and merit further research. Differences at baseline and between consistent and inconsistent attendees also merit further study because of the unknown effects of these differences and other undetected covariables. Another limitation is that particular results may be beyond covariate adjustment and influenced by the type of participant in the treatment groups or the treatment itself. For example, computer-prompt participants showed increased computer use, which could be an unintended consequence or a byproduct of the intervention itself.

Our novel and complex study design extends the literature, because it evaluated a multilevel (ie, individual, physical, social–environmental, and organizational) intervention, an important shift from previous single-focused interventions (2,9). This study has other strengths, including a randomized controlled design, a racially and ethnically diverse study sample, objective measurements of physiologic outcomes, and a 6-month trial period. The racially and ethnically diverse study sample is important, because research predicts that the workforce will become increasingly diverse (24). Previous research has reported cultural and ethnic differences related to cardiovascular risk factors and other health indicators (25); we found racial and ethnic differences in baseline measures but no differences in study outcomes.

Previous research has documented that job strain, defined as the ratio of job demands to job control, is a risk factor for elevated blood pressure and is related to lower representation in surveys and research (26,27). Therefore, we recommend that future studies include measures of job strain and comprehensive assessments of psychosocial stressors so that employees experiencing high levels of job strain will be represented in study samples. Additionally, to better understand differences between consistent and inconsistent attendees, thorough analyses are recommended of management’s commitment and support for active work breaks versus devaluing such breaks in favor of meeting work demands (7).

Workplace Booster Breaks may mitigate major barriers to physical activity, including lack of time, concerns about neighborhood safety, lack of social support, and costs of equipment, workout attire, and gym membership (28). The challenge is to implement workplace interventions with sufficient fidelity and dose. Management support and commitment are essential to provide incentives and to communicate to employees that sustained participation is critical and expected. If practiced routinely during the workday, Booster Breaks can achieve the twin goals of promoting physical activity and reducing sedentary behavior. Given this potential, further research to replicate, refine, and enhance the effects of the Booster Break program is warranted.
